# Non-Equilibrium Block Copolymer Self-Assembly Based Porous Membrane Formation Processes Employing Multicomponent Systems

**DOI:** 10.3390/polym15092020

**Published:** 2023-04-24

**Authors:** Lieihn Tsaur, Ulrich B. Wiesner

**Affiliations:** 1 Department of Materials Science and Engineering, Cornell University, Ithaca, NY 14853, USA; 2 Kavli Institute at Cornell for Nanoscale Science, Cornell University, Ithaca, NY 14853, USA

**Keywords:** block copolymers, self-assembly, non-equilibrium, multicomponent, hierarchical structures, SNIPS

## Abstract

Porous polymer-derived membranes are useful for applications ranging from filtration and separation technologies to energy storage and conversion. Combining block copolymer (BCP) self-assembly with the industrially scalable, non-equilibrium phase inversion technique (SNIPS) yields membranes comprising periodically ordered top surface structures supported by asymmetric, hierarchical substructures that together overcome performance tradeoffs typically faced by materials derived from equilibrium approaches. This review first reports on recent advances in understanding the top surface structural evolution of a model SNIPS-derived system during standard membrane formation. Subsequently, the application of SNIPS to multicomponent systems is described, enabling pore size modulation, chemical modification, and transformation to non-polymeric materials classes without compromising the structural features that define SNIPS membranes. Perspectives on future directions of both single-component and multicomponent membrane materials are provided. This points to a rich and fertile ground for the study of fundamental as well as applied problems using non-equilibrium-derived asymmetric porous materials with tunable chemistry, composition, and structure.

## 1. Introduction

Materials design is inherently susceptible to performance tradeoffs. For structural materials, increasing material strength often requires sacrificing toughness and flexibility [1]. For porous polymer membrane materials that, e.g., help address progressively more complex separation problems as well as the growing issue of global water scarcity [2], increasing flux through a material generally comes at the expense of selectivity [3]. Nature assembles multiple building blocks, each with their own structure and function, into asymmetric and hierarchical structures to address such tradeoffs [4,5,6]. Bone is a mineralized composite consisting of inorganic hydroxyapatite nanocrystals deposited along organic collagen fibrils to form lamellae, whereby self-assembly (SA) of the latter defines the framework and spatial constraints of the former, and the spatial heterogeneities are responsible for the superior mechanical properties [7]. The respiratory system is hierarchically constructed such that a breath of air travels down the trachea into the main bronchi of the lungs, through the even smaller bronchiole tubes into the capillary-covered alveoli to allow high flux while maximizing the surface area for O_2_/CO_2_ exchange. Engineering such hierarchical, asymmetric structures with compositional heterogeneity calls for non-equilibrium structure formation processes employing multicomponent systems.

For filtration membranes, these structural requirements can be achieved by superimposing block copolymer (BCP) self-assembly onto the extensively used, industrially proven non-solvent-induced phase separation (NIPS, or phase inversion) method, a process now referred to as SNIPS [8]. Traditional homopolymer-based phase inversion membranes exhibit an asymmetric support structure, with nanometer to micrometer porosity, topped by a dense, disordered skin layer [9,10]. Equilibrium BCP self-assembly generates a variety of periodically ordered nanostructures (e.g., cubic lattices of spherical micelles, hexagonal lattices of cylindrical micelles, lamellae, co-continuous cubic structures, etc.), where the resulting morphology is determined by the relative volume fractions of the individual blocks [11]. Non-equilibrium SNIPS harnesses this inherent nanometer (typically 10–100 nm) length scale of BCP microphase separation to generate a thin (~100–400 nm) layer of dense and periodically ordered, uniformly sized pores mechanically supported by the phase-inverted substructure, simultaneously achieving high resolution and throughput. First demonstrated by Peinemann et al. using an amphiphilic poly(styrene-*b*-4-vinylpyridine) (SV) system [12], this process was subsequently successfully applied to other diblock copolymer systems [13,14,15,16,17]. Phillip et al. showcased the benefits of utilizing multiblock (>2) polymer systems to generate SNIPS membranes by elucidating the functional (in this case, mechanical) improvements exhibited by triblock terpolymer poly(isoprene-*b*-styrene-*b*-4-vinylpyridine) (ISV) derived membranes compared to their SV diblock membrane counterparts [18]. Other efforts involving triblock terpolymer derived SNIPS membranes support this trend [19,20,21,22]. In all cases, the general SNIPS procedure remains the same. The casting dope, consisting of a BCP dissolved in a binary or ternary solvent system, is drawn across a substrate using a doctor blade. The resulting film is allowed to evaporate for tens of seconds, during which a concentration gradient develops along the film normal, predominantly due to the evaporation of the more volatile solvent (usually tetrahydrofuran (THF)). Near the film surface, upconcentration is sufficient to drive BCP SA, creating a periodically ordered skin-like layer of only about 100 nm thickness. The evaporation is halted by plunging the film into a non-solvent (usually deionized (DI) water) bath, which precipitates the BCP, effectively freezing the concentration gradient as a graded, asymmetric membrane structure.

**Figure 1:**
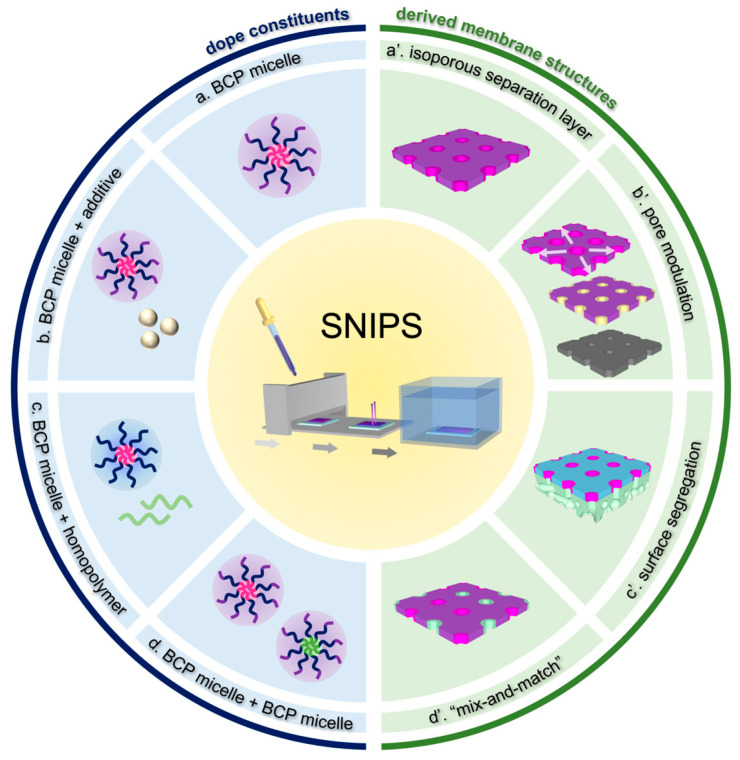
Schematic overview of concepts presented in this review. (**left**) Types of dope constituents (a,b,c,d) and (**right**) corresponding derived membrane structures (a’,b’,c’,d’) resulting from (**center**) self-assembly and non-solvent-induced phase separation (SNIPS) based processes. Colored structures refer to organic/polymer materials and gray color represents purely inorganic structures.

Despite the successful implementation of the SNIPS process in a vast number of BCP systems, our understanding of the complicated interplay of non-equilibrium processes during membrane formation remains incomplete. Müller and Abetz presented an overview of theoretical approaches and experimental results regarding non-equilibrium processes in membrane formation, with a particular focus on homopolymer and diblock copolymer systems [23]. The current review, with emphasis on work conducted by the Wiesner group, starts by summarizing our current understanding of the top surface structural evolution during single-component triblock terpolymer (ISV) SNIPS membrane formation. ISV SNIPS membranes are a model system for structural study because the integration of the PI block not only expands the range of BCP molar masses that are mechanically stable enough for membrane formation, but also gives rise to an additional layer of structural hierarchy in the form of mesoporous walls due to terpolymer phase separation within the substructure [24]. Thereafter, we explore how multicomponent systems involving the addition of a small-molecule additive, homopolymer, or second BCP to the original BCP casting dope can impart new functionality and utility to the derived materials. This will include the addition of inorganic materials precursors, opening pathways to translate the structural control achievable with SNIPS to inorganic materials and membranes. Figure 1 provides a schematic overview of the concepts presented in this review.

## 2. Single-Component ISV SNIPS Membranes

### 2.1. General Overview of Structure and Properties

**Figure 2:**
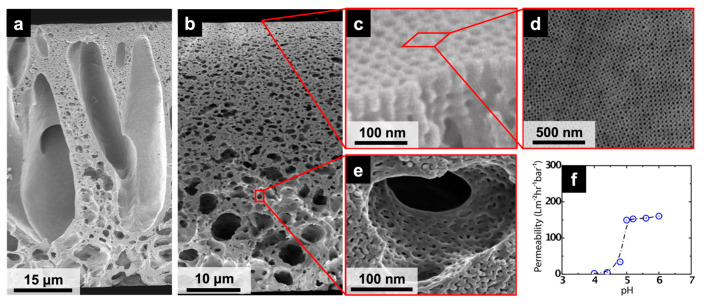
Structure and performance characteristics of ISV SNIPS membranes. (**a**) Finger-like substructure. (**b**) Sponge-like substructure. (**c**) Cross-sectional view of separation layer, showing the interconnected cubic pore network. (**d**) Membrane top surface revealing high-density, periodically ordered pores as well as typical grain boundaries between differently ordered cubic lattices. (**e**) Mesoporous pore walls of the macroporous substructure. Regions of interest outlined in red not to scale. (**f**) Performance of ISV membrane under varying pH conditions. (**b**,**d**,**e**) Adapted from [33], Copyright 2022, with permission from Elsevier. (**c**,**f**) Adapted with permission from [18]. Copyright 2011 American Chemical Society.

A typical ISV SNIPS membrane consists of a ~100 nm thick separation layer decorated with 2D square packed pores (Figure 2c,d) that range from ~10 to 20 nm in diameter (as determined by SEM analysis), putting these membranes in the ultrafiltration (UF) regime [25]. Supporting the ordered separation layer is a typically <100 μm thick graded asymmetric substructure, where pore sizes increase with distance from the separation layer. By adjusting casting parameters such as polymer concentration, solvent composition (particularly the ratio of 1,4-dioxane (DOX) to THF), evaporation time, and non-solvent bath temperature, the substructure can be tuned from finger-like (Figure 2a) to sponge-like (Figure 2b) [26]. An additional level of structural hierarchy is present towards the bottom of the membrane, where the walls of the larger pores themselves become mesoporous (Figure 2e), likely due to terpolymer phase separation [24]. ISV SNIPS membranes with the above structures to date have been successfully derived from terpolymers containing volume fractions of ~20–30% PI, ~50–60% PS, and ~10–30% P4VP [26,27,28]. ISV SNIPS membrane performance tests revealed that hydraulic permeability was a strong function of pH, with a substantial drop in permeability occurring between pH 4 and 5 (Figure 2f), providing evidence that the P4VP block (pKa ~ 4.6 [29]) decorated the pore walls. Below its pKa, the P4VP block becomes protonated, and chain extension due to charge repulsion and increased solubility in the aqueous buffer leads to pore closure. The P4VP brushes can be post-functionalized with, e.g., methyl iodide (or 1,3-propane sultone) to confer the membrane with positively (or negatively) charged pore walls for charge-based separations [30,31], or hydrolase enzymes to confer enzymatic recognition capability, providing a route to biocatalytic “skin-like” materials [32].

### 2.2. Elucidation of Structure Formation Mechanisms

**Figure 3:**
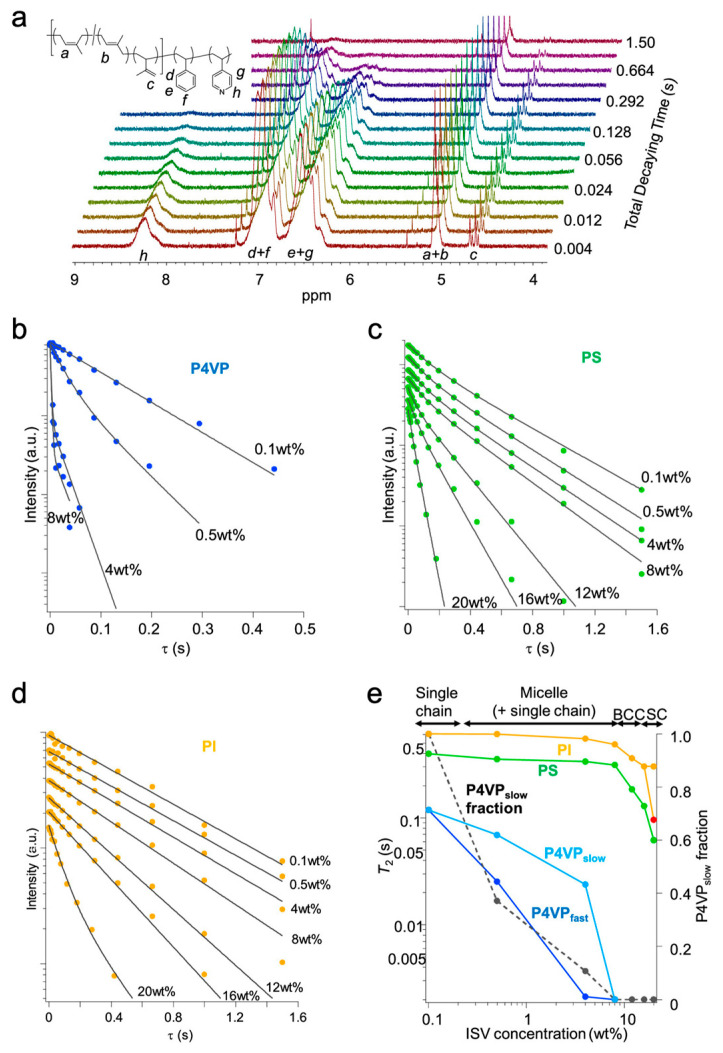
*T*_2_ relaxation analysis for various concentrations of ISV90 in 4DOX/6THF (by wt.). (**a**) Representative NMR spectra displaying increasing *T*_2_ relaxation with increasing decay times in the NMR experiments. *T*_2_ relaxation behavior of (**b**) P4VP, (**c**) PS, and (**d**) PI blocks at different ISV concentrations as observed for specific proton signals of each component, as shown in the inset of panel (**a**). Measured signal intensities (y-axes) are presented on a log scale. Intensities were translated along the longitudinal axis for ease of view. (**e**) *T*_2_ dependence of each block on ISV concentration: (blue) fast relaxing component of P4VP; (teal) slow relaxing component of P4VP; (green) PS; (orange) slow relaxing component of PI; (red) fast relaxing component of PI; and (black) fraction of slow relaxing component of P4VP representing unimers in solution. Reprinted with permission from [28]. Copyright 2020 American Chemical Society.

While the direct influence of SNIPS casting parameters (e.g., polymer concentration, evaporation time, etc.) and ISV molar mass on final ISV membrane surface structure, substructure, and/or performance has been carefully studied [26,27,34,35], improvements in our fundamental understanding of the structural formation mechanism over the past decade have been slow. Despite the general agreement that the microphase separation-driven BCP micelle formation is responsible for the observed uniform surface pore structure upon phase inversion, even for diblock copolymer-based SNIPS membranes, details regarding micelle structure (i.e., which polymer blocks constituted the corona vs. core) and the micelle-to-pore evolution remained under debate [36,37,38]. In the case of ISV SNIPS membranes derived from the ternary ISV/DOX/THF system, the P4VP and PI blocks were initially presumed to respectively constitute the micelle corona and core based on Hansen solubility parameter calculations minimizing unfavorable polymer–solvent enthalpic interactions [18]. Subsequent ^1^H nuclear magnetic resonance (NMR) spin–spin relaxation time (*T*_2_) studies on various concentrations (0.1 to 20 wt. %) of ISV90 (90 kg/mol, 22:51:27 volume fractions) in 4DOX/6THF by wt. (representative spectra in Figure 3a) instead revealed that above the critical micelle concentration (CMC), ISV micelles preferentially adopt PI/PS coronas and segregated P4VP cores to minimize thermodynamically unfavorable solvent interactions [28]. *T*_2_ relaxation times reflect molecular motion, with slower-moving molecules exhibiting shorter *T*_2_. In the current context, core block constituents are expected to display smaller *T*_2_ relative to their corona and single-chain (unimer) counterparts because chain entanglements and solvent exclusion largely restrict core motion, allowing magnetization to relax faster. Between 0.1 and 0.5 wt. % ISV, the P4VP *T*_2_ drastically decreased (relaxation of magnetization became faster) and transformed from mono-exponential to biexponential (Figure 3b). This observation can be rationalized by ISV going through its CMC in this concentration range, leading to a coexistence of unimers and micelles with P4VP cores at 0.5 wt. %. As the corresponding PI and PS *T*_2_ relaxation behaviors did not exhibit substantial changes in this concentration range akin to single chains (compare Figure 3b with Figure 3c,d), these blocks were assigned to the motionally less-restricted micelle corona. This picture (i.e., PI and PS constituting the micelle corona, and P4VP constituting the micelle core) is consistent with that obtained from SV and PS-*b*-poly(2-vinylpyridine) (PS-*b*-P2VP; S2V) copolymer studies where the shorter hydrophilic P4VP or P2VP blocks form the micelle cores [39,40]. Although the concentration of unimers remains fixed beyond the CMC, their relative fraction becomes imperceptible in the semi-dilute (10–20 wt. %) regime (see dashed line in Figure 3e). The reliance of SNIPS membrane pore formation on top surface micelle assembly suggests that suitable casting solution concentrations should fall within the semi-dilute regime.

**Figure 4:**
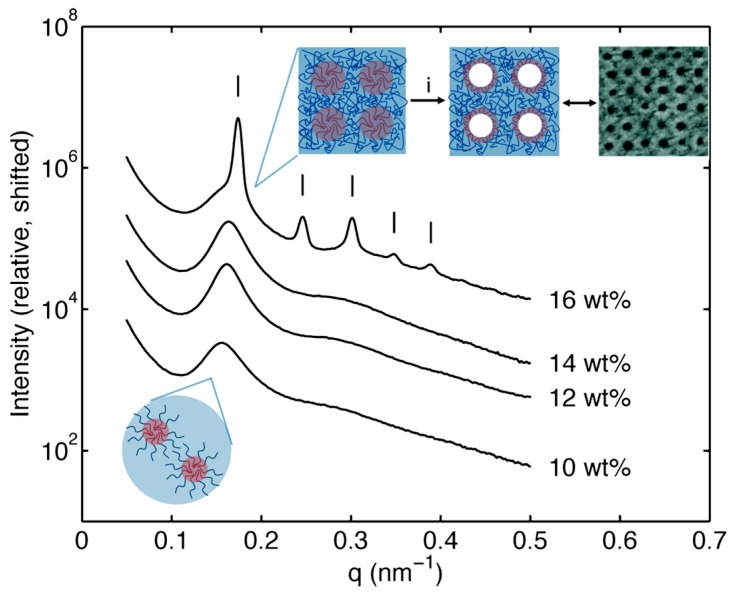
Quiescent ISV terpolymer solution SAXS profiles at various concentrations in 7DOX/3THF (by wt.). Tick marks for 16 wt. % trace are consistent with a BCC lattice. Insets depict disordered (bottom left) and periodically ordered (top right) ISV micelle (navy: PI, PS; red: P4VP) solution structures. Arrow (i) in top right inset corresponds to the SNIPS phase inversion step that precipitates the BCP. This step quickly causes the less swollen PS matrix to freeze into its glassy state, not giving the system enough time to fully relax, thus leaving the more swollen P4VP chains unable to adapt, resulting in open pores. Adapted with permission from [8]. Copyright 2012 American Chemical Society.

Dorin et al. first established, using ISV and SV polymers, that screening block copolymer solutions via small-angle X-ray scattering (SAXS) could help predict surface pore structure and provide an upper limit for casting dope concentration [8]. While the current review will focus on the ISV system, readers are referred to Marques et al. for a detailed follow-up study on the SV system [41]. At concentrations slightly above typical casting solution concentrations, quiescent solutions comprising ISV polymer in a 7DOX/3THF (by wt.) binary solvent system showed higher-order reflections consistent with ISV micelles sitting on a body-centered cubic (BCC) lattice (Figure 4) [27]. During SNIPS, preferential solvent evaporation from the top surface allows ISV concentrations to quickly reach this threshold for micelle lattice formation. Subsequent replacement of solvent by non-solvent during phase inversion causes solvent-swollen polymer chains to collapse, forming open pores arranged in a 2D square lattice (see top right inset in Figure 4). Pore formation upon phase inversion signifies a non-equilibrium state of the system, as complete solvent evaporation (allowing the system to reach equilibrium) would result in a dense, non-porous film. At the time, the 2D square-packed pores in the final membrane were thought to be geometrically consistent with the cubic (BCC) lattice observed in the more concentrated quiescent solution. However, whereas a BCC lattice could give rise to square pore packing if the (001) plane were oriented parallel to the membrane surface, careful inspection of scanning electron microscopy (SEM) images depicting the three to four micelle layers comprising the ISV membrane top separation layer instead suggested a three-dimensional simple cubic (SC) pore network (Figure 2c) [18]. This discrepancy motivated subsequent in situ studies to elucidate the evaporation-induced surface structural evolution.

**Figure 5:**
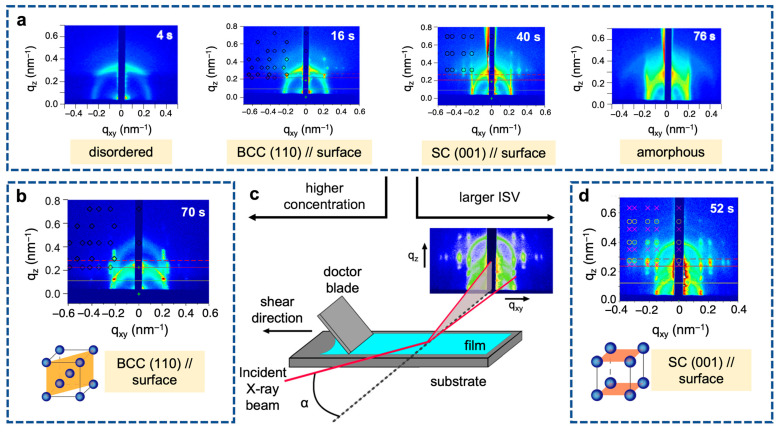
In situ GISAXS experimental setup and patterns. GISAXS patterns of (**a**) 16 wt. % ISV43, (**b**) 20 wt. % ISV43, and (**d**) 10 wt. % ISV91 films evaporated for the indicated times. Expected spots marked for the indicated lattices and in-plane orientations. (**c**) Schematic of in situ GISAXS experimental setup. Adapted with permission from [42]. Copyright 2016 American Chemical Society.

A custom-built automated doctor blading setup was used to conduct in situ grazing incidence small-angle X-ray scattering (GISAXS) measurements at the Cornell High Energy Synchrotron Source (CHESS) D1 beamline (Figure 5c). Doctor-bladed films derived from a 16 wt. % ISV43 (43 kg/mol) terpolymer solution (a typical dope concentration) showed transitions from (1) disorder to order, (2) order to order, and (3) order to amorphous surface structures during SNIPS-relevant time scales (<100 s) (Figure 5a) [42]. GISAXS pattern analysis revealed that the initially disordered film first transitioned to a structure consistent with the (110) plane of a BCC lattice oriented parallel to the surface. At a later evaporation time, however, the GISAXS pattern was best fitted with a (001) in-plane oriented SC lattice before the surface structure subsequently evolved into an amorphous, short-range ordered state. When a more concentrated ISV43 solution-derived film was probed, order onset occurred at a much later time (46 s vs. 10 s), and the only ordered surface structure observed was the BCC (110) plane (Figure 5b). In contrast, when using a typical dope concentration of a larger ISV91 (91 kg/mol) terpolymer, the corresponding film only displayed disordered → SC (001) → amorphous structural transitions (Figure 5d). These subsequent experiments suggest that the slower kinetics associated with solutions derived from either a higher polymer concentration (higher solution viscosity) or a larger molar mass terpolymer (lower micelle mobility) prevent observation of the order-to-order transition. For both ISV terpolymers, the quiescent solution SAXS- and in situ GISAXS-obtained first-order peak positions (q*)—which reflect the characteristic morphological length scales—were similar. Likewise, the SC lattice parameters (*a* = 2π/q*) derived from the 16 wt. % ISV43 and 10 wt. % ISV91 solution films agreed well with the lattice parameters (pore-to-pore distances) of the final membrane top surfaces (28 vs. 26 nm and 42 vs. 45 nm for ISV43 and ISV91, respectively).

**Figure 6:**
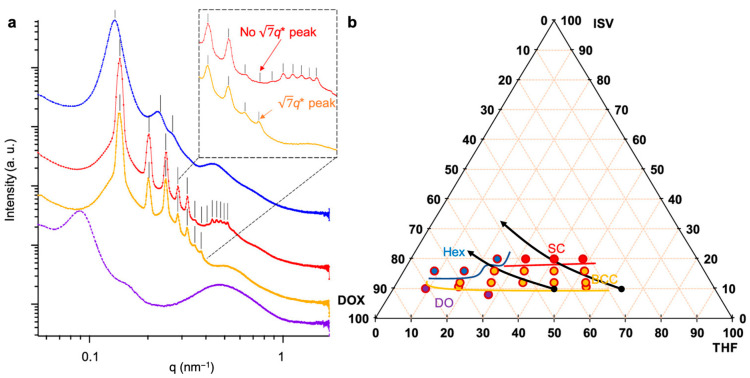
Ternary phase diagram of ISV/DOX/THF as elucidated by quiescent solution SAXS experiments. (**a**) Representative SAXS profiles consistent with (blue) hexagonal cylinders, (red) SC lattice, (yellow) BCC lattice, and (purple) disordered (DO) micelles. BCC and SC lattices can be distinguished by the existence or nonexistence of a peak at q = √7q* for BCC and SC, respectively (see inset). (**b**) ISV/DOX/THF ternary phase diagram derived from SAXS experiments. Black arrows denote two predicted evaporation induced solution composition trajectories (based on Raoult’s law calculations) from 0 to 120 s of evaporation. Adapted with permission from [28]. Copyright 2020 American Chemical Society.

Whereas in situ observance of SC lattice symmetry (Figure 5a,d) corroborated the 4-fold square lattice symmetry and three-dimensional SC pore network exhibited in top surface and cross-sectional SEM images of the final membranes, respectively (Figure 2c,d), the origin of the BCC-to-SC lattice transition remained unclear. Stegelmeier et al. first described the solvent evaporation-induced compositional changes in an S2V/*N,N*-dimethylformamide (DMF)/THF system, where the relative volume fractions of S2V and higher boiling point DMF increase with evaporation time [40]. Given that THF has a lower boiling point than DOX, the ternary (ISV/DOX/THF) system becomes increasingly ISV- and DOX-rich with increasing evaporation time, deviating from the initial 7DOX/3THF (by wt.) solvent ratio that prior solution SAXS studies (yielding predominantly BCC solution structures) were based on. Coupled with the in situ GISAXS BCC-to-SC transition observed for the 16 wt. % ISV43 system at longer evaporation times (Figure 5a), this suggested that the root cause of the order-to-order transition may be polymer and/or DOX concentration related. To test this hypothesis, Hibi et al. mapped out the ISV/DOX/THF ternary phase diagram (Figure 6b) in the region relevant for SNIPS membrane formation by performing solution SAXS experiments on a range of ISV solution concentrations (8–20 wt. %) and DOX/THF ratios (9:1–4:6) (representative SAXS traces in Figure 6a) [28]. Beyond the disorder-to-order transition (~10 wt. %) and up to 16 wt. % ISV, micelles preferentially formed BCC lattices over the range of DOX/THF ratios tested. Above 16 wt. % ISV, higher DOX fraction solutions (above 7DOX/3THF by wt.) displayed hexagonal cylinder (Hex) lattices, whereas lower DOX fraction solutions displayed SC lattices. The structural differentiator between these two pathways was hypothesized to be whether the PI block segregated from the PS block in the micelle corona to minimize unfavorable solvent interactions. DOX is the better solvent for the PI block. Therefore, if, in highly concentrated solutions (20 wt. %), the DOX solvent fraction is not high enough (i.e., lower than 7DOX/3THF by wt.), PI block segregation into spherical domains could give rise to two interpenetrating SC lattices consisting of P4VP and PI micelle cores shifted by half the cubic lattice diagonal akin to a CsCl SC lattice, which would be consistent with solution SAXS analysis. For higher DOX solvent fractions, micellar coalescence at higher concentrations would instead give rise to a Hex lattice. To verify this hypothesis, we turn again to the prior ^1^H NMR *T*_2_ relaxation experiments conducted in the dilute CMC regime as well as in semi-dilute solutions (8–20 wt. % ISV in 4DOX/6THF by wt.) [28]. Around 10 wt. % ISV (at the disorder-to-order transition), when micelles start adopting BCC lattices (according to solution SAXS), free solvent absorption by the polymer leads to significant corona–corona overlap. This restricted corona motion is reflected by the shortened *T*_2_ observed for the corona-forming PI and PS blocks in this concentration range (8–12 wt. % ISV; see orange and green traces in Figure 3e). Between 16 and 20 wt. % ISV, the PI relaxation behavior switches from mono- to biexponential, whereas that of PS remains mono-exponential (compare Figure 3c,d). This is consistent with PI segregating from PS to form PI micelles, which experience further restricted mobility (shortening *T*_2_; see red datapoint in Figure 3e). This observation lines up with the BCC → SC transition observed via solution SAXS, substantiating the hypothesis that this transition is driven by evaporation-induced PI segregation.

### 2.3. Perspectives on Single-Component ISV SNIPS: The Inverted Designer Cycle and Templating Inorganic Materials

**Figure 7:**
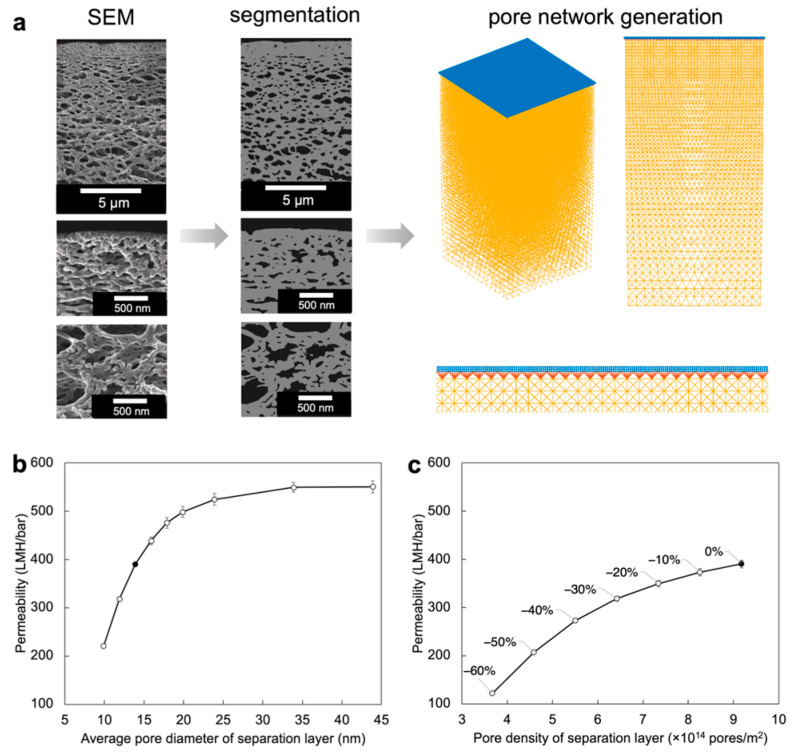
Pore network generation and sensitivity analysis. (**a**) Process of generating a 3D pore network model from a series of 2D SEM micrographs with different magnifications. Effects of changes in (**b**) average pore diameter and (**c**) separation layer pore density on simulated permeability. Black markers denote reference permeability value (390.68 ± 7.90 LMH/bar) simulated based on (**b**) a pore size distribution of 13.90 ± 1.72 nm and (**c**) pore density of 9.18 × 10^14^ pores/m^2^, respectively. Adapted from [33], Copyright 2022, with permission from Elsevier.

Despite advances made in our understanding of the structural formation mechanisms in ISV SNIPS membranes, there has not yet been a set of experiments where quiescent solution SAXS, in situ GISAXS, and the final membrane top surface structure as observed by SEM all tell a consistent story. With the help of theoretical studies [43], in situ studies encompassing the final phase inversion stage of SNIPS membrane formation could help inform what further structural changes occur upon polymer precipitation, possibly completing the structural puzzle. Thus far, SNIPS membrane research has predominantly been “bottom-up” in the sense that a BCP is synthesized, optimal membrane formation parameters are identified, and subsequently, a series of rejection experiments inform what the membrane properties—including permeability and solute rejection behavior—are (i.e., BCP → membrane → property/application cycle). The ultimate goal in understanding all the structural components constituting ISV SNIPS membranes as well as their flow and separation properties is to reverse the SNIPS membrane formation cycle. This would enable defining, based on a desired permeability and separation profile, the necessary SNIPS membrane structure, which in turn would define the molecular architecture of the BCP submitted to the SNIPS process (i.e., inverted “designer” cycle of property/application → membrane → BCP). While the Hagen–Poiseuille equation can relate separation layer characteristics (e.g., thickness, surface porosity, pore size, and pore tortuosity) to theoretical permeability, the flow resistance stemming from the disordered integral support layer often leads to substantial overestimation of permeability [44]. Three-dimensional (3D) SNIPS membrane reconstructions can provide valuable information about layer-by-layer porosity and pore size distribution in the substructure that would enable more accurate flow modeling [45,46], but such techniques are often time- and cost-intensive. Therefore, as a first step towards making SNIPS membrane modeling more accessible, Riasi et al. recently delineated the hierarchical asymmetric pore structure of two ISV membranes derived from different sized terpolymers using a series of 2D SEM images with varied resolutions (Figure 7a) [33]. Flow simulated using a novel stochastic pore network model led to computed absolute permeabilities in good agreement with experimental results. Sensitivity analysis revealed that simulated permeability was more sensitive to changes in pore diameter than pore density (Figure 7b,c). Further studies, perhaps involving machine learning (ML) [47], could help elucidate how fabrication conditions and other structural parameters (e.g., pore connectivity, macrovoids in the substructure, and separation layer thickness) contribute to the experimentally observed property profiles, bringing us one step closer to realizing inverted designer cycles in the making of SNIPS-based UF membranes.

**Figure 8:**
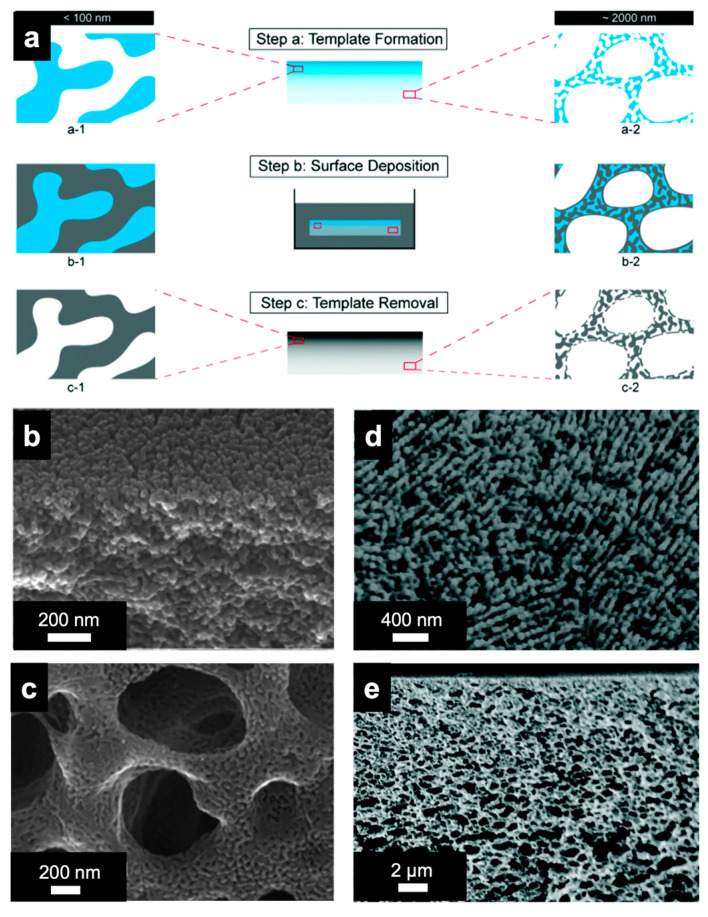
Schematic representation of the preparation and final structure of porous inorganic CGMs. (**a**) Steps involved in the preparation of CGMs (**center**) and enlarged portions of mesopores near the top of the film (**left**) and mesoporous macropore walls near the bottom of the film (**right**) associated with each step. Polymer component in blue, void space in white, and backfilled carbon precursors or metals in gray. SEM characterization of CGM-Carbon: (**b**) cross-section of the top skin layer showing small carbon pillars originating from the cylindrical pores of the polymer template and (**c**) bottom surface demonstrating accessible macro- and mesopores. SEM characterization of CGM-Ni: (**d**) top surface and (**e**) cross-section. Reproduced from [48] with permission from the Royal Society of Chemistry.

The high surface area and highly accessible pores—a combined result of the densely porous top separation layer and asymmetric meso- to macroporous substructure—make non-equilibrium (ISV) SNIPS-derived asymmetric UF membranes attractive materials for numerous other applications, including catalytic conversions as well as energy conversion and storage. Practically, however, such materials require properties typical of carbons, metals, and oxides (e.g., high temperature stability, electrical and thermal conductivity, mechanical strength, etc.). As such, using ISV SNIPS membranes as soft templates for these hard materials could converge the necessary structure–property requirements. To that end, Gu et al. developed protocols to template the hierarchically porous ISV SNIPS membrane structure into graded porous carbon and metal (oxide) materials, referred to as Cornell graded materials (CGMs) [48]. Templating was achieved by depositing the desired material either via direct immersion in a precursor solution (carbon) or by electroless plating (Ni, Cu), followed by a series of thermal treatments for template removal and transformation into the final material (Figure 8a). SEM characterization of the final CGMs showed faithful templating of the ISV membrane structure, from the mesoporous top separation layer down to the mesoporous walls of the macroporous substructure (Figure 8b–e). The intermediate polymer–metal hybrid materials could be of interest for applications requiring both soft and hard material properties. Additionally, given the versatility of this soft templating method, nanostructured CGMs could serve as a runway for the formation of other functional materials classes, following in the footsteps of thin film analogues [49].

## 3. Multicomponent Approaches to SNIPS-Derived Asymmetric Porous Materials

Efforts to realize SNIPS-derived membrane materials with the desired structural or chemical attributes have predominantly taken one of two general directions, each with its own opportunities and challenges: (I) Introducing novel polymer chemistries, either by adding an additional block to a known SNIPS-compatible BCP or by designing an entirely new BCP, requires sophisticated synthetic techniques and extensive casting parameter optimization; (II) Post-fabrication functionalization techniques can rely on chemically modifying the pores of existing optimized SNIPS systems, but their challenge lies in achieving the desired functionalization on industrially relevant timescales (i.e., compatible with roll-to-roll processing) [30,31,50,51,52]. The former changes the identity of the material subjected to the casting process, while the latter only changes the identity of the final membrane material. Taking inspiration from the tradition of polymer blending [53,54,55,56,57,58,59], a convenient middle ground would be to simply blend additional components into the casting dope along with the SNIPS-compatible BCP. The following subsections demonstrate the utility afforded by incorporating additives, homopolymers, or even a second BCP into the original BCP casting dope.

### 3.1. CNIPS-Derived Membranes from BCP plus Additives in the Dope

To date, the combination of BCP-additive co-assembly with non-solvent-induced phase separation (CNIPS) has been harnessed to synthesize purely organic, hybrid organic–inorganic as well as purely inorganic asymmetric membrane materials structure-directed by ISV terpolymer self-assembly.

#### 3.1.1. CNIPS-Derived Membranes from Organic Additives

**Figure 9:**
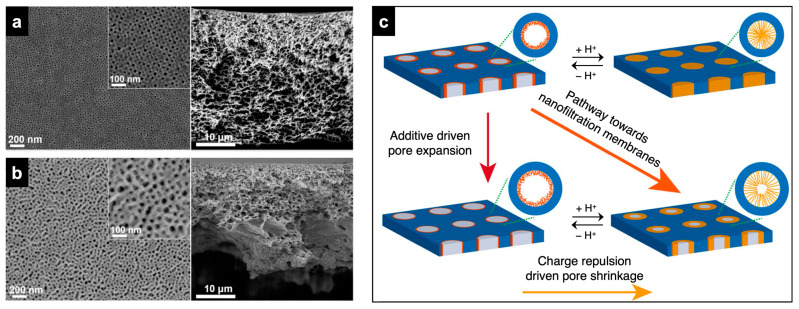
Glycerol-induced SNIPS/CNIPS membrane structural changes and derived pathway towards nanofiltration membranes. Top surface and cross-sectional SEM characterization of membranes derived from solutions with (**a**) m_glycerol_/m_ISV_ = 0 (i.e., pure ISV) and (**b**) m_glycerol_/m_ISV_ = 0.40. (**c**) Schematic representation of pH-responsive behavior of (**top**) a pure ISV SNIPS membrane top surface layer exhibiting quasi “on-off” behavior as a function of pH and (**bottom**) an ISV–glycerol CNIPS membrane top surface layer that retains a finite pore size even at low pH due to glycerol-induced pore swelling, generating a nanofiltration membrane. Adapted with permission from [60]. Copyright 2015 American Chemical Society.

Gu and Wiesner demonstrated that by incorporating glycerol, a non-toxic organic additive, into the ISV casting dope up to m_glycerol_/m_ISV_ = 0.40, they could increase the pore size of the final membrane from 23 nm to 48 nm while maintaining fairly uniform pore sizes (compare Figure 9a,b) [60]. Aside from the initial preparation of the multicomponent casting dope, the casting → evaporation → phase inversion steps of the SNIPS process were unchanged. Glycerol’s three -OH groups preferentially interact with the pore-forming P4VP block situated in the micelle core, swelling it in the solution state. Upon phase inversion, the swollen P4VP chains contract, and the water-soluble glycerol is washed out, increasing pore size. The P4VP–glycerol interactions in solution also allowed the concentration of ISV in the solution to be cut by more than 50% (m_ISV_/m_solvent_: 0.14 → 0.06), which is of interest for cost-driven industrial applications. Similar effects of organic additive-induced pore size and solution viscosity modulation were observed in diblock copolymer systems. The addition of carbohydrates (e.g., α-cyclodextrin, saccharose) [61] or -OH/-COOH functionalized molecules (e.g., rutin, 9-anthracenemethanol) [62] to SV-containing solutions led to increased porosity, more pronounced hexagonal surface morphology, and increased membrane permeability. Yang et al. utilized poly(ethylene glycol)s with a range of molar masses (550 to 20,000 g/mol) to modulate the pore size and permeability of PS-*b*-poly(acrylic acid) SNIPS membranes [63]. In the ISV–glycerol system, the combination of glycerol-driven pore expansion with the protonation-driven chain extension of P4VP under acidic (pH < 4.6) conditions allowed pore size reductions down to 5 nm, bridging ultrafiltration and nanofiltration with the same membrane (Figure 9c). Organic additive incorporation allows access to a large range of SNIPS membrane pore sizes using a single BCP, rather than requiring the synthesis of multiple, different molar mass BCPs [27,64] or extensive post-functionalization steps [65,66,67].

#### 3.1.2. CNIPS-Derived Organic–Inorganic Hybrid Membranes from Inorganic Additives

**Figure 10:**
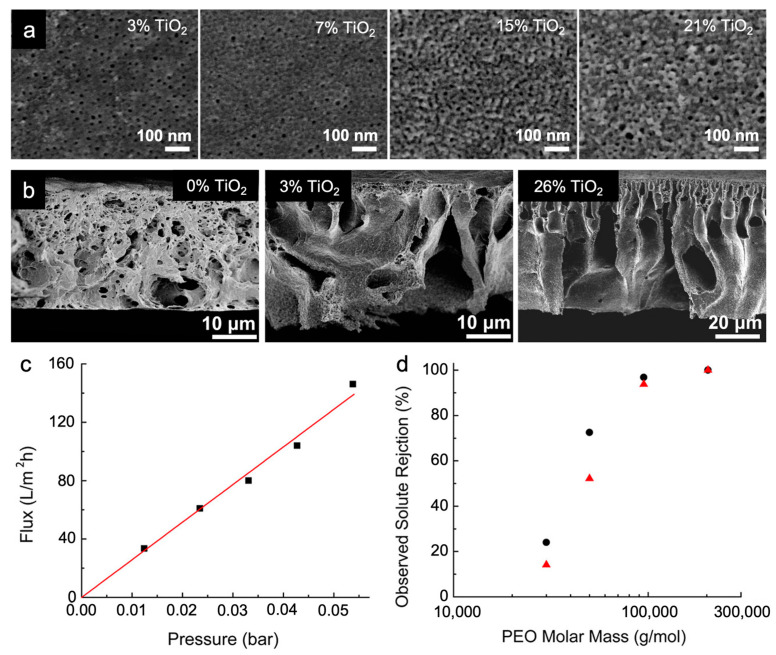
Organic–inorganic CNIPS-derived hybrid membrane structure and performance. SEM micrographs of (**a**) top surfaces and (**b**) cross-sections of membranes cast from solutions containing different percentages of TiO_2_ (i.e., mass of TiO_2_ introduced into the casting solution over total mass of ISV and TiO_2_; as indicated). (**c**) DI water flux of organic–inorganic hybrid membrane with 15 wt. % retained TiO_2_ at various transmembrane pressures. (**d**) Molecular weight cut-off curves for two hybrid membranes (15 wt. % TiO_2_ retained) cast from identical conditions at ~0.02 bar (black) and ~0.03 bar (red) applied pressure. Adapted with permission from [69]. Copyright 2013 American Chemical Society.

In the prior example of an ISV-additive system, the approach was designed so that the additive would wash out upon phase inversion, imparting no chemical changes to the final membrane itself. However, CNIPS can also be used to permanently incorporate additives into the final membrane, conveying new chemical functionality. One example is the incorporation of inorganic nanoparticles (NPs) to create organic–inorganic hybrid membranes that merge the flexibility and processability of organics with the stability and chemical activity of inorganics in a “one-pot” process. Titanium dioxide (TiO_2_) is a particularly attractive candidate for separation applications as its high hydrophilicity and antimicrobial behavior in the crystalline state could help combat membrane fouling [68]. Gu et al. prepared TiO_2_ sol NPs via a hydrolytic sol–gel route employing titanium tetraisopropoxide (TTIP) precursors and added different amounts of TiO_2_ sol solution to existing ISV/DOX/THF solutions. Using this method, up to 15 wt. % TiO_2_ was incorporated into the final hybrid membrane (vs. 21 wt. % TiO_2_ initially added to the casting solution), as inferred from thermogravimetric analysis (TGA) [69]. These amounts (wt. % additive added and retained) are an order of magnitude higher than those of (1) prior inorganic salts (0.15–2.5 wt. % of the entire solution), added with the primary intention of micelle stabilization [36,70], most of which may be washed away upon immersion in the non-solvent bath or (2) graphene oxide nanosheets (<1.5 wt. % with respect to BCP) added to increase fouling resistance [71]. The following structural changes were observed in hybrid membrane structure as TiO_2_ loading increased: (1) surface pore morphologies changed from straight and circular to tortuous and network-like, consistent with selective swelling of P4VP domains by TiO_2_ sol NPs due to preferential interactions (Figure 10a); (2) substructures transitioned from sponge-like to finger-like (Figure 10b), suggesting that the hydrophilic Ti-OH capped TiO_2_ sol NPs facilitate solvent–non-solvent (DOX/THF and DI water) exchange, leading to faster ISV precipitation. Hybrid membranes (15 wt. % TiO_2_ retained, network-like surface pore structure) displayed an order of magnitude increase in permeability relative to purely organic membranes prepared under similar conditions (3200 vs. ~150 LMH/bar), while maintaining size-based selectivity (Figure 10c,d). The molecular weight cut-off (at 90% rejection) for these hybrid membranes was ~90 kg/mol PEO (Figure 10d), comparable to the ~100 kg/mol PEO cut-off of a pure membrane made from the same ISV polymer [27]. The high inorganic loading and high permselectivity expand the functional potential of such CNIPS-derived organic–inorganic hybrid membranes. This was further demonstrated by Zhang et al., who added TiO_2_ sol NPs to a casting dope containing a novel diblock copolymer with a hydrophobic majority block and an amphiphilic minority block (as opposed to the typical hydrophobic majority block(s) + hydrophilic minority block combination). The derived organic–inorganic hybrid membranes (1) had highly macroporous bottom surfaces conducive to higher permeability, (2) showed excellent mechanical stability (up to 2.9 bar applied pressure) and antifouling performance, and (3) could be post-functionalized to yield swollen polyelectrolytic pore walls compatible with nanofiltration of anionic molecules [72].

#### 3.1.3. CNIPS-Derived Inorganic Membranes from Inorganic Additives

##### CNIPS-Derived Asymmetric Carbons

For applications where the poor chemical resistivity of ISV may pose a problem, a purely inorganic membrane that maintains the ordered top separation layer and asymmetric, hierarchical pore substructure of an ISV SNIPS membrane is desirable. As described earlier, this can be achieved by using an ISV SNIPS membrane soft template, and backfilling with the target inorganic precursor. Ideally, however, this would be achieved using a “one-pot” CNIPS procedure rather than necessitating numerous post-processing steps. In 2015, Hesse et al. subjected a one-pot ISV and phenol formaldehyde resols solution (the simultaneous method, vide infra) to the CNIPS process and used subsequent heat treatment to crosslink the resols and remove the polymer. While this yielded asymmetric, hierarchically porous carbon materials, a periodically ordered top surface typical of SNIPS-derived materials was not achieved [73]. As optimal performance in electrochemical energy storage (EES) devices rests upon balancing the high surface area [74] afforded by a periodically ordered top separation layer and well-defined structural micro- and mesoporosity with the enhanced transport and surface accessibility [75] realized by the hierarchical asymmetric pore substructure, this warranted further study.

**Figure 11:**
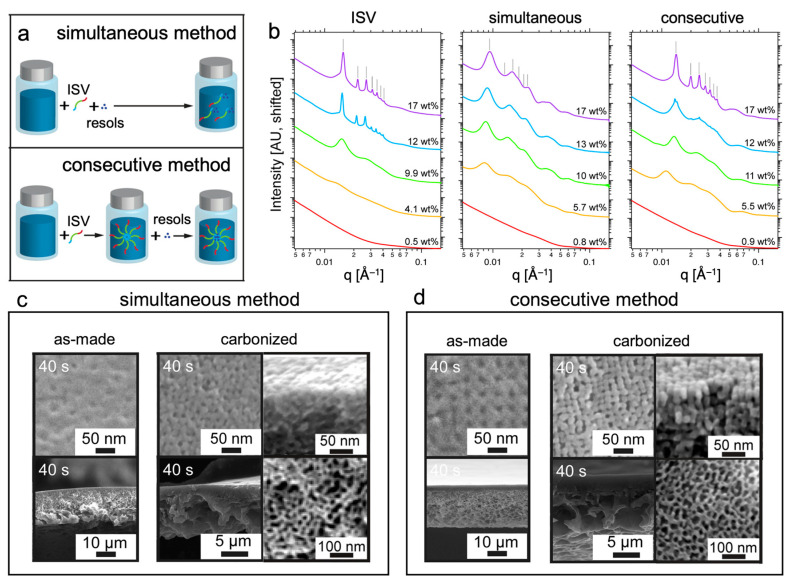
Pathway complexity observed for non-equilibrium CNIPS approach to asymmetric carbon materials. (**a**) Schematic comparing the simultaneous and consecutive methods of solution preparation. (**b**) SAXS patterns of parent ISV, simultaneous method ISV + resols (2:1 ISV:resols by wt.), and consecutive method ISV + resols (2:1 ISV:resols by wt.) exhibiting improved order for the latter mixture, similar to parent ISV. Simultaneous and consecutive method concentrations reflect ISV + resols. Tick marks indicate expected peak positions for a BCC lattice relative to the observed primary peak. SEM micrographs comparing the top surface and cross-sectional structures of as-made and carbonized membranes synthesized using (**c**) simultaneous method and (**d**) consecutive method. For the carbonized membranes, higher magnification micrographs of the top surface cross-sectional view and the substructure mesoporosity are provided, again showing substantially improved periodic order of the top surface for the consecutive method. Adapted with permission from [76]. Copyright 2021 American Chemical Society.

In 2021, Hesse et al. demonstrated that when preparing casting solutions, the formation of ISV micelles prior to resols addition was key to obtaining ordered top surfaces in the as-made hybrid CNIPS membranes and heat-treatment-derived carbon materials [76]. Casting solutions prepared from (1) ISV powder + solvated resols (the “simultaneous method”) and (2) ISV micelles + solvated resols (the “consecutive method”) (Figure 11a) were probed via quiescent solution SAXS and in situ GISAXS. While solution SAXS profiles for the consecutive method were, albeit showing slightly larger lattice spacings (e.g., 63 nm for 17 wt. % ISV + resols vs. 57 nm for 17 wt. % ISV), qualitatively similar to those of pure ISV/DOX/THF (i.e., displaying cubic micelle lattice formation at higher solution concentrations), those for the simultaneous method reflected much larger structures (e.g., 96 nm lattice spacings for 17 wt. % ISV + resols) and lacked well-defined long-range periodic order (Figure 11b). This was hypothesized to be because, in the simultaneous case, early hydrogen bonding between resols and the P4VP block upon ISV dissolution prevents efficient incorporation of P4VP into homogenously sized micelle cores, leading to non-uniformly sized larger micelles that are structurally and kinetically hindered from forming well-defined cubic lattices. In situ GISAXS yielded results consistent with solution SAXS, namely that casting solutions prepared via the consecutive method went through the disordered → ordered → amorphous transitions with increasing evaporation time typical of pure ISV casting solutions. In contrast, scattering patterns from the simultaneous method were dominated by polycrystalline structures without well-defined long-range periodic order. For the consecutive case, the patterns of the intermediate periodically ordered structure were consistent with an SC lattice ((001) in-plane direction) for casting solution concentrations between 8 and 13 wt. % and a BCC lattice ((110) in-plane direction) for a 15 wt. % casting solution. The latter observation is likely because the higher solution viscosity prevents the segregation of PI and PS in the micelle corona responsible for the BCC → SC transition (vide supra). Compared to the ideal casting conditions for pure ISV SNIPS membranes (room temperature (~20 °C) substrate and ~40% relative humidity), the ISV-resols CNIPS hybrid membrane surface structure was optimal at higher substrate temperatures (30 °C) and lower relative humidity (<30%). As-made membranes (following 30 s precipitation in nonsolvent bath) from the consecutive method showed square-packed surface pore structures with low pore–matrix contrast under SEM—a result of the hydrogen-bonded resols filling the P4VP pore space (Figure 11d, left). Carbonized materials—achieved via a series of heat treatments to crosslink the resols, remove the ISV template, and carbonize the remaining material—displayed top surfaces consisting of square-packed pillars corresponding to the original pore space, with largely reduced pillar-to-pillar distance (compared to pore-to-pore distance of as-made membrane) due to shrinkage following ISV matrix removal (Figure 11d, right). Surface porosity now stems from the interstitial space of the pillared matrix. Regardless of preparation method (consecutive or simultaneous) and surface order (presence or lack thereof), carbonized materials retained their asymmetric structure and mesoporosity amidst shrinkage (Figure 11c,d). Surprisingly, nitrogen sorption isotherms analyzed using the Brunauer–Emmett–Teller (BET) method showed that the simultaneous-method-derived disordered carbonized membrane materials had higher porosities than the corresponding consecutive-method-derived ordered materials (1322 vs. 1024 m^2^ g^−1^). This carbonized membrane architecture is well-suited for energy storage and conversion applications (vide infra).

##### CNIPS-Derived Asymmetric Nitrides

**Figure 12:**
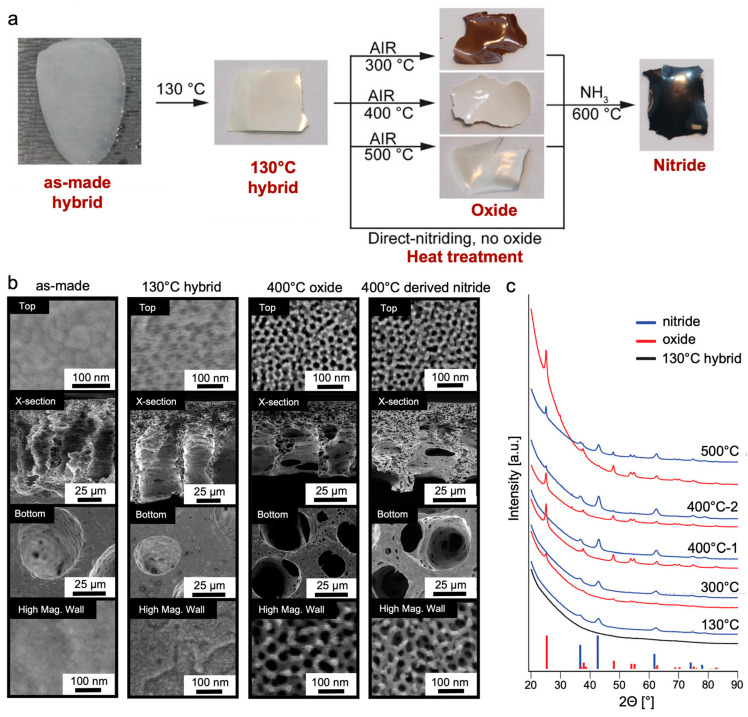
CNIPS thermal processing steps and structural characterization of derived materials. (**a**) Schematic of thermal processing steps involved in oxide and nitride formation from as-made organic–inorganic hybrid membranes. (**b**) Representative SEM micrographs of asymmetric CNIPS materials after every major processing step. From left to right: as-made, 130 °C hybrid, 400 °C oxide, 400 °C oxide-derived nitride. From top to bottom: top surface, asymmetric cross-section, bottom surface, and high-magnification image of macropore walls in the substructure. (**c**) XRD of asymmetric materials obtained from the four pathways to TiN (blue) and their corresponding precursors (hybrid in black, oxides in red). Red tick marks correspond to the expected peak positions and relative intensities of a tetragonal crystal system of anatase TiO_2_ (I4_1_/*amd*, space group #141, ICSD #01-070-7348). Blue tick marks correspond to the expected peak positions and relative intensities for cubic TiN (F*m*$\bar{3}$*m*, space group #225, ICSD #00-038-1420). Traces from two separate samples oxidized at 400 °C (and subsequently nitrided) are shown, demonstrating sensitivity to thermal processing. Adapted with permission from [77]. Copyright 2022, John Wiley & Sons, Inc.

Similar efforts were made to elucidate the structure–processing–property relationships of titanium nitride (TiN) materials derived from the ISV-TiO_2_ hybrids described earlier [77]. TiN is an attractive alternative material for aqueous electrochemical double-layer capacitors (EDLCs) due to its high conductivity, acid stability, and higher voltage stability [78]. A series of thermal treatment protocols (Figure 12a) applied to the as-made hybrid material heated to 130 °C revealed that (1) the presence of an intermediate oxide step, and (2) the temperature (300–500 °C) at which the oxide was formed, determined the porosity profile and phase purity (mixed-phase anatase + TiN or phase-pure TiN) of the final membrane material. Furthermore, tuning the CNIPS evaporation time of the ISV-TiO_2_ hybrids allowed modulation between finger-like and sponge-like substructures reflected in the final heat-treated inorganic material. The following general structural observations were made: (1) as-made hybrids exhibited closed top surfaces and asymmetric cross-sections with non-mesoporous macropore walls that extended to the bottom surface, (2) heat treatment to 130 °C revealed under-developed hexagonally packed pores decorating the top surface, while the substructure walls remained non-mesoporous, and (3) polymer decomposition upon oxidizing or nitriding at elevated temperatures gave rise to clearly hexagonally ordered porous top surfaces and mesoporous substructure walls (Figure 12b). X-ray diffraction (XRD) characterization revealed that phase-pure TiN materials could be achieved by direct nitridation (TiN-d) of the hybrid material, or by going through an intermediate oxide formation step conducted at 300 °C (TiN-300) or 400 °C (TiN-400). Oxide formation at 500 °C prevented complete subsequent nitridation, as evidenced by the retained crystalline oxide peaks in XRD (Figure 12c). BET surface areas were 105, 178, and 90 m^2^ g^−1^ for TiN-d, TiN-300, and TiN-400, respectively, demonstrating the pathway dependence of the obtained structures and associated properties.

##### Electrical Double Layer Capacitors from CNIPS-Derived Asymmetric Inorganic Materials

**Figure 13:**
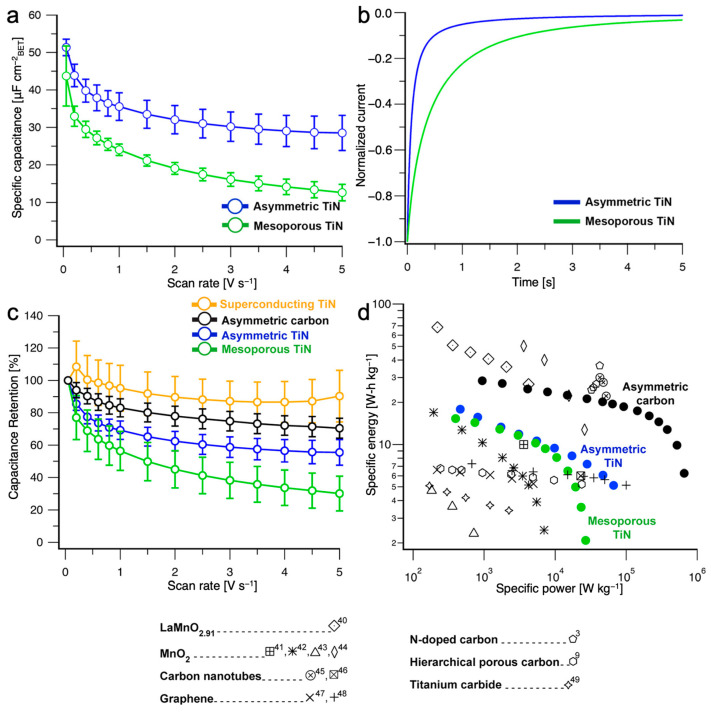
Electrochemical characterization and capacitor performance benchmark of gyroidal mesoporous TiN and CNIPS-derived asymmetric TiN and carbon materials. (**a**) Scan rate dependence of specific capacitance for asymmetric TiN (blue) and mesoporous TiN (green) showing improved surface accessibility in asymmetric TiN. (**b**) Chronoamperometry starting from the open-circuit voltage to 0.01 V vs. RHE (reversible hydrogen electrode) showing enhanced ion diffusion in asymmetric TiN. (**c**) Scan rate dependence of capacitance retention for superconducting asymmetric TiN, asymmetric carbon, asymmetric TiN, and gyroidal mesoporous TiN. (**d**) Ragonne plot comparing energy storage performance of gyroidal mesoporous TiN, asymmetric TiN, and asymmetric carbon to current state-of-the-art materials reported in the literature. Error bars in (**a**,**c**) represent standard deviations from three independent trials. All results were collected in Ar-saturated 0.1 mol L^−1^ HClO_4_. Adapted with permission from [81]. Copyright 2020 American Chemical Society.

Energy conversion and storage devices seek to converge high energy density (e.g., in capacitors afforded by the large surface areas of meso- and microporous materials) with high power density (e.g., in capacitors realized by rapid accessibility of pore structures) [79,80]. However, simultaneous accomplishment of both from a structural standpoint is non-trivial as, e.g., high pore surface area comes with small pores, and thus with slow accessibility. To demonstrate how the degree of structural hierarchy (the coexistence of micro-, meso-, and/or macropores), well-defined mesoporosity everywhere, and non-equilibrium-induced structural asymmetry of the CNIPS-derived TiN and carbon materials contribute to pore accessibility and ion diffusion rates, Hesse et al. conducted cyclic voltammetry (CV) in aqueous 0.1 M HClO_4_ over a range of scan rates (50 mV s^−1^ to 5 V s^−1^) [81]. Capacitive response per internal surface area was obtained via normalization of measured current by BET surface area. The effects of structural asymmetry were elucidated by comparing the high-scan-rate capacitive retention of asymmetric CNIPS-derived TiN-400 and a feature-matched (in terms of thickness, pore size distribution, and specific surface area) homogeneous gyroidal mesoporous TiN (i.e., an equilibrium structure formation process based mesoporous material with maximal pore accessibility via co-continuous cubic gyroidal morphology). Even though the three-dimensional alternating gyroid structure provides the mesoporous TiN with highly accessible, interconnected mesopores [82], its capacitive loss at high scan rates was almost twice as high (~70% vs. 44% loss for mesoporous TiN and asymmetric TiN-400, respectively; Figure 13a). This effect was attributed to the faster ion diffusion in asymmetric TiN—supported by chronoamperometry experiments (Figure 13b)—demonstrating the perks of asymmetric pore structures derived from non-equilibrium CNIPS formation processes. Despite having the lowest BET surface area (compared to TiN-d and TiN-300, vide supra), TiN-400 had the highest specific capacitance at all scan rates tested [77], suggesting that the effects of increased pore accessibility outweighed those of reduced surface area in mass transport. Moreover, 70% high-scan-rate capacitive retention for activated CNIPS-derived graphitic carbon materials (Figure 13c) demonstrated that structural asymmetry enhances mass transport even in the presence of substantial microporosity (<2 nm pores). Calculated power densities at competitive energy densities (of a single electrode using a three-electrode half-cell configuration) for both asymmetric materials were substantially higher than state-of-the-art materials: 28.2 kW kg^−1^ at 7.3 W-h kg^−1^ for asymmetric TiN, and 287.9 kW kg^−1^ at 14.5 W-h kg^−1^ for asymmetric graphitic carbon (Figure 13d). Superconducting asymmetric TiN materials with higher electrical conductivity relative to original asymmetric TiN materials (but two orders of magnitude lower than bulk TiN) demonstrated 90% high-scan-rate capacitive retention (vs. 56% retention for non-superconducting asymmetric TiN; Figure 13c), suggesting that these measurements reflect material rather than structural (asymmetry) limitations. This leaves room for further material improvement to probe the limits of pore accessibility in these graded asymmetric materials.

### 3.2. Surface SNIPS (S^2^NIPS) Derived Membranes from BCP plus Homopolymer in the Dope

**Figure 14:**
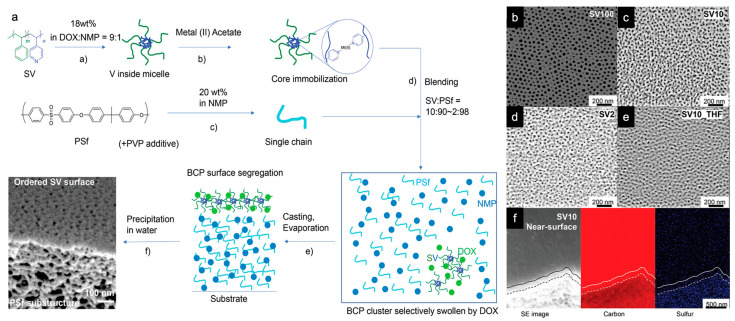
Depiction of surface SNIPS (S^2^NIPS) procedure and successful implementation thereof. (**a**) Schematic of one-step S^2^NIPS process toward a dual-layer membrane consisting of an SV BCP self-assembly based porous separation layer sitting atop an asymmetric PSf substructure cast from a blended solution of the two polymers into an UF membrane using SNIPS. Top surface SEM micrographs of membranes comprising (**b**) pure SV, (**c**) 10 wt. % SV, and (**d**) 2 wt. % SV all cast from a DOX/NMP solution, and (**e**) 10 wt. % SV cast from a THF/NMP solution. For S^2^NIPS membranes, the denoted weight fraction of SV is relative to the total polymer weight (SV + PSf + PVP) in solution. (**f**) EDX mapping of 10 wt. % SV derived membrane cross-section near the top surface, showing carbon-rich (SV-rich) separation layer and sulfur-rich (PSf-rich) substructure. Solid lines indicate the surface/cross-section boundary, whereas dashed lines highlight the position of the separation layer/substructure (BCP/PSf) interface. Adapted with permission from [86], Copyright 2021, John Wiley & Sons, Inc.

The prior section focused on the co-assembly of an ISV terpolymer with (in)organic additives that preferentially partition into the pore-forming P4VP domains to augment, chemically modify, or template the ISV pore structure. While similar effects can be achieved by adding a homopolymer in place of an additive, e.g., by blending in P4VP homopolymer to selectively increase the ISV SNIPS membrane pore diameter [18], homopolymers can themselves form phase inversion membranes [83], which means successful membrane formation is not contingent on their selective segregation into a single block copolymer domain. One interesting question that arose was whether, in a single process (i.e., involving some form of industrially scalable NIPS) and by careful design of BCP plus homopolymer and solvent constituents in the polymer dope, segregation of a thin BCP SA-derived mesoporous separation layer atop an asymmetrically porous homopolymer substructure (i.e., a dual-layer membrane) could be achieved. This would drastically reduce the amount of BCP required, thereby cutting cost, and making BCP-based SNIPS membranes more commercially viable. Dual-layer membranes that reduce the amount of BCP needed have recently been achieved by casting highly diluted (1 wt. %) BCP solutions onto polyacrylonitrile (PAN) supports [84] or by co-casting the BCP with a commercially available homopolymer (e.g., polysulfone (PSf)) [85]. The former still resulted in a 0.9–2.6 μm BCP layer (compared to the typically ~100 nm thick separation layers commonly obtained from SNIPS), whereas the latter required two consecutive casting steps. In stark contrast, Hibi and Wiesner recently reported on proof-of-principle experiments demonstrating that by using two solvents with sufficient differences in their surface energy, each of which preferentially adsorbs one of the polymer constituents (BCP or homopolymer), the constituent selectively swollen by the lower surface energy solvent can be segregated to the surface on SNIPS-relevant timescales (<100 s). This created dual-layer membranes via a single casting step, a process termed surface SNIPS (S^2^NIPS) (Figure 14a) [86]. This non-equilibrium dual-layer formation process is driven by differences in solvent surface energies instead of the more commonly encountered thermodynamic drivers such as minimizing polymer surface energy or maximizing entropy by placing more chain ends at the surface. It can thus be used to segregate two polymer constituents with similar surface energies regardless of their number of chain ends. Applying this technique to an SV diblock copolymer plus PSf homopolymer system—with DOX or THF as the SV selective solvent and *N*-methyl-2-pyrrolidone (NMP) as the PSf selective solvent—complete SV surface coverage could be achieved down to 2 wt. % SV (relative to total polymer weight in solution). This was confirmed by SEM (Figure 14b–e) and accompanying energy-dispersive X-ray spectroscopy (EDX) mapping of carbon and sulfur (Figure 14f). Blending in poly(vinylpyrrolidone) (PVP), which is miscible with PSf in solution and dissolves away upon phase inversion in a water bath, led to increased substructure macropore size, thereby increasing membrane permeability without affecting surface segregation. Following casting parameter optimization, proof-of-principle first-generation dual-layer membranes outperformed a highly optimized commercial BioMax membrane (with similar pore size) in both throughput and resolution. Crucial to successful implementation of S^2^NIPS were (1) P4VP core immobilization to prevent micelle flipping in NMP-rich (e.g., >90 NMP/10 DOX by wt.) solutions, (2) using two solvents whose surface energy differences were large enough, and (3) not adding more DOX or THF than can be taken up by SV clusters, as excess DOX/THF may distribute across both polymer phases, preventing effective segregation. Surface segregation persisted even when films were allowed to evaporate until dry prior to phase inversion, making this strategy applicable to general film technologies.

### 3.3. “Mix-and-Match” Derived Asymmetric Membranes from Mixtures of Chemically Distinct BCPs in the Dope

**Figure 15:**
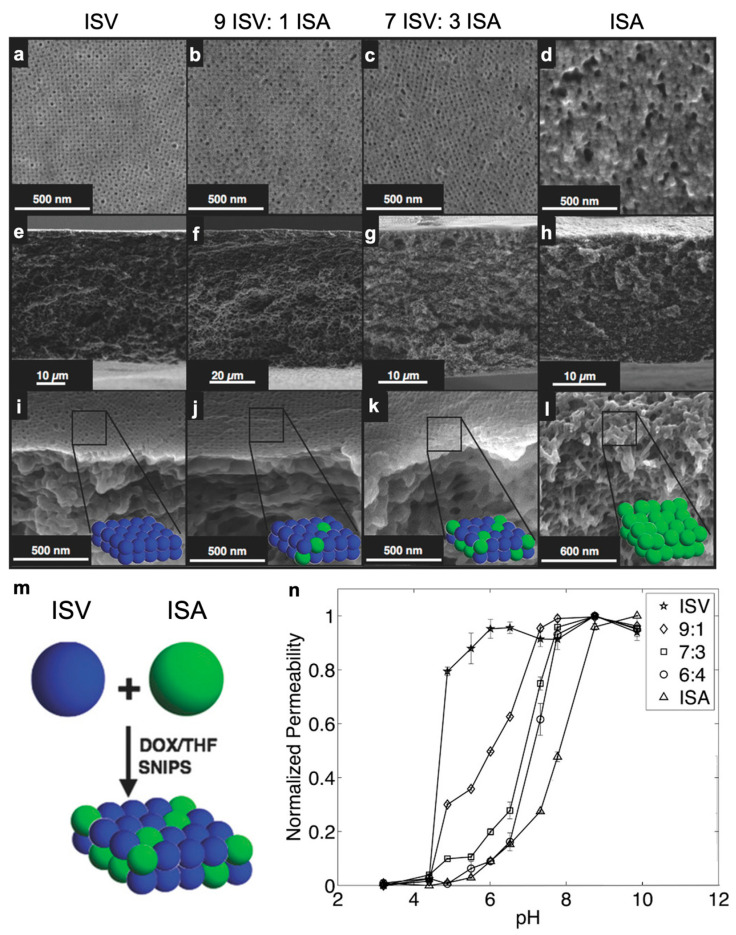
Structure and properties of membranes derived from blends of two chemically distinct triblock terpolymers. SEM characterization of surface structures (**top row**), cross-sections (**second row**), and areas close to the surface (**third row**) of (**a**,**e**,**i**) a pure ISV membrane, (**b**,**f**,**j**) a 9:1 ISV:ISA blended membrane, (**c**,**g**,**k**) a 7:3 ISV: ISA blended membrane, and (**d**,**h**,**l**) a pure ISA membrane. The ISA membrane was cast from a 7DMF/3THF solution. All other membranes were cast from 7DOX/3THF solutions. (**m**) Simplified depiction of blended membrane formation from two chemically distinct micelle populations, with ISV and ISA micelles in blue and green, respectively. Micelle distribution in the final membrane top surface layer is displayed for illustrative purposes only and not a result of detailed structural investigations. (**n**) Normalized permeability for pure (ISV, ISA) and blended (9:1, 7:3, and 6:4 blends of ISV:ISA) membranes at various pH values. Error bars: Standard deviations from three replicate measurements. All ratios depicted are weight ratios. Adapted with permission from [87], Copyright 2016, John Wiley & Sons, Inc.

The idea of controlling the distribution of two chemically distinct polymer domains during the evaporation process opens the door to realizing multifunctional SNIPS membranes without post-modification. If, in a blended polymer dope, the homopolymer component described above is replaced by a second BCP, preferably one that has a different pore-forming (micelle core forming) block, then co-assembly of the two micelle populations should result in a separation layer with two different pore chemistries (see schematic depiction in Figure 15m), provided that (1) both populations preferentially adsorb the same solvent and (2) do not undergo chain exchange on the SNIPS timescale (<100 s). For the first time, Li et al. successfully demonstrated this concept using ISV and poly(isoprene-*b*-styrene-*b*-dimethylaminoethyl methacrylate) (PI-*b*-PS-*b*-PDMAEMA, ISA), two terpolymers that differ only in their pore-forming blocks [87]. The chemically identical corona blocks (PI + PS) ensure that the above-mentioned solvent-driven surface segregation effects do not apply, while the room-temperature processing conditions, relatively large molar masses (~100 kg/mol), and relatively concentrated BCP casting solutions (15 wt. %) make chain exchange unlikely [88]. ISV and ISA were separately dissolved in 7DOX/3THF by wt.—pre-forming the individual ISV and ISA micelles—before the solutions were subsequently mixed for 10 min and subjected to the regular SNIPS protocol. It should be noted that this blending method is fundamentally different from earlier work by Radjabian and Abetz [89], where two differently sized, but otherwise chemically identical, SV copolymers (e.g., SV1 and SV2) were simultaneously dissolved in solvent, forming mixed micelles that contain both SV1 and SV2. This is also fundamentally different from work by Yu et al. in which micellized SV polymer was blended with non-micellized PS-*b*-poly(acrylic acid), and unimers of the latter inserted themselves into the micelles of the former to increase pore density and hydrophilicity [90]. For up to 30 wt. % ISA (relative to total polymer mass), the resulting membranes showed a well-ordered ~200 nm separation layer and a sponge-like substructure typical of SNIPS membranes (compare Figure 15a,e,i with Figure 15b,f,j and Figure 15c,g,k). Blending provides a method for tuning in a new pore chemistry without the extensive optimization of a new terpolymer system such as ISA, which by itself under the conditions tested did not form a well-ordered top surface (Figure 15d,h,l). Given that P4VP and PDMAEMA have different pKa values (4.6 and 7.8 [91], respectively) at which protonation-driven chain extension and pore closure occurs, the presence of the two pore chemistries could be confirmed by examining permeability behavior across a range of pH values. As expected, the blended membranes exhibited intermediate pH-responsive behavior, with higher ISA content leading to pore closure onset at higher pH values (Figure 15n), demonstrating proof-of-principle. Absolute permeabilities of the 9:1 and 7:3 ISV:ISA (by wt.) blended membranes in the “open” state (i.e., pH 10; 300–600 LMH/bar) were between those of the pure membranes (~250 and ~850 LMH/bar for ISV and ISA, respectively). This “mix-and-match” approach opens the door for more complex multifunctional materials realized purely by the design of the casting dope BCP constituents.

### 3.4. Perspectives on Multicomponent SNIPS

The immense playground for multicomponent, multifunctional materials synthesized from derivatives of the original SNIPS protocol (e.g., CNIPS, S^2^NIPS, or “mix-and-match”) is still largely unexplored. Recently, the separations community has trended towards synthesizing ultrathin separation layers from inherently porous organic materials and subsequently transferring them onto polymeric or anodic alumina oxide (AAO) supports [92,93,94,95]. While these cases have realized record-breaking resolution and throughput, ensuring defect-free transfer and good adhesion between layers, especially on a larger scale, is non-trivial. Applying the concepts of S^2^NIPS here would eliminate the need for such subsequent processing steps and be more conducive to scale-up.

Although originally developed for separations science, asymmetric, hierarchical structures with well-defined mesopores everywhere resulting from the non-equilibrium SNIPS formation process with triblock terpolymers such as ISV can be translated to applications beyond those relating to energy storage and conversion detailed here. The preferential interactions between the P4VP nitrogen and other transition metal oxide derived sol NPs (e.g., Ta_2_O_5_ or SrTiO_3_) could be leveraged to structure-direct hierarchical, asymmetric materials boasting high surface area and mass transport rates. This may not only be advantageous for electrochemical device applications in energy storage and conversion, as demonstrated for asymmetric carbon and nitrides in an EDLC (vide supra), but also as novel supports for catalytic or photocatalytic conversions. To expand the library of organic and inorganic materials that can be subjected to CNIPS, additional molecular interactions, e.g., hydrophobic interactions or click chemistry, could be harnessed by exploring different structure-directing BCPs.

When given enough time to reach equilibrium, multicomponent materials (e.g., polymer blends) generally exhibit phase segregated structures, with one component typically enriched at the material–air interface due to thermodynamic considerations, thus largely limiting the structural arrangements that can be achieved. The beauty of a non-equilibrium formation process is the ability to puppeteer the distribution of individual components using environmental parameters and subsequently freeze-in the desired structure before the system has equilibrated. Extrapolating from the “mix-and-match” approach described earlier, an additional layer of structural control is afforded by engineering, e.g., the interactions between different micelle populations from highly attractive to highly repulsive (e.g., using electrostatic interaction potentials), and everything in between, thereby controlling—in the case of a separation membrane—the relative distribution of pore chemistries in the selective top layer. Drawing inspiration from colloidal building blocks, BCP micelle analogues could be designed to assemble into crystalline lattices or “solid solution”-type structures [96]. Taking the “mix-and-match” approach to the next level, one could also combine the micellar building block approach with CNIPS, where each micelle population structure directs a distinct (in)organic additive to yield a structurally interconnected composite material after the polymer matrix has been thermally removed. All these chemical permutations may be achievable simply through clever dope designs, simultaneously rendering them compatible with roll-to-roll fabrication processes, and thus facilitating rapid translation into industrial scale-up.

## 4. Conclusions

The superposition of BCP self-assembly and industrially scalable non-equilibrium phase inversion (SNIPS) provides a versatile platform to generate graded, asymmetric materials that simultaneously exhibit properties previously thought to be mutually exclusive. Combining asymmetric pore structures with mesopores everywhere in the resulting materials overcomes typical trade-offs observed in porous materials obtained from equilibrium approaches (e.g., high permeability and high selectivity; high surface area and high pore accessibility). This review showcased examples of the application of this emerging platform to multicomponent dopes. By essentially keeping the main processing steps the same, but moving to cleverly designed multicomponent dopes, the range of accessible asymmetric porous structures and compositions can be substantially broadened. Multicomponent dopes also provide access to areas of applications well beyond the original scope of ultrafiltration, including nanofiltration, electrochemical devices for energy storage and conversion, and potentially as supports for various catalytic and photocatalytic conversions. We have highlighted approaches to multifunctional porous organic materials, but also expanded the library of SNIPS-derived materials from porous organics to organic–inorganic hybrids and even pure inorganics. Resulting materials classes with asymmetric pore structure ranged from polymers, semiconductors, and metals all the way to superconductors. In almost all cases, compositional variations were achieved without compromising the structural benefits afforded by the combination of BCP self-assembly with non-solvent-induced phase separation based asymmetric pore structure formation. We hope we have been able to demonstrate that such advanced multicomponent-based non-equilibrium approaches open a realm of possibilities with substantial academic as well as industrial promise.

## Data Availability

All new data generated for this study (Figure 2a) is available within the review article. All other data presented in this review is available in the referenced literature.
